# Effects of Beraprost with or without NOS Inhibition on Plasma Aldosterone and Hemodynamics in Healthy Cats

**DOI:** 10.3390/vetsci11040155

**Published:** 2024-03-30

**Authors:** Takumi Matsuura, Aritada Yoshimura, Ryuji Fukushima

**Affiliations:** 1 Toray Industries, Inc., Tokyo 103-8666, Japan; 2 Animal Medical Center, Tokyo University of Agriculture and Technology, Tokyo 183-8509, Japan; 3 Animal Medical Emergency Center, Tokyo University of Agriculture and Technology, Tokyo 184-8588, Japan

**Keywords:** beraprost, feline, chronic kidney disease, aldosterone, NOS

## Abstract

**Simple Summary:**

Beraprost is a stable prostacyclin analogue that has been shown to be effective and safe for the treatment of chronic kidney disease (CKD) in cats. However, the pharmacological effects of beraprost on the renin-aldosterone (RA) axis and hemodynamics in cats were largely unknown, and the prescription of beraprost has been often limited to feline patients with related disorders. This study in healthy cats demonstrated that the clinical dose of beraprost for feline CKD reduces plasma aldosterone, which is reversed by nitric oxide synthase (NOS) inhibition, without changes in the plasma renin and hemodynamics. These findings may help in understanding the pharmacological mechanism, and also the clinical safety of beraprost in the treatment of feline CKD.

**Abstract:**

Objectives: The aim of the study was to evaluate the hemodynamic and RA system effects of the oral administration of the clinical dose of beraprost for feline CKD in healthy cats, and also to examine whether NOS inhibition reversed them. Methods: A placebo-controlled pharmacological sequential design study was carried out to assess the plasma aldosterone and renin concentrations (PAC and PRC), blood pressure, heart rate, and exploratorily to estimate renal plasma flow (RPF) and renal vascular resistance (RVR) with simplified methods. Results: Beraprost reduced PAC when compared to the placebo (*p* < 0.05); this was reversed when NOS inhibitor NG-nitro-L-arginine methyl ester (L-NAME) was added to the beraprost treatment (*p* < 0.01). No differences in the PRC or hemodynamic parameters were detected between beraprost and the placebo. The correlation ratios (η^2^) showed opposite relationships between beraprost and the added L-NAME effects on PAC, mean blood pressure (MBP), heart rate, estimated RPF (*p* < 0.001), estimated RVR (*p* < 0.01), and PRC (*p* < 0.05). Conclusions: In healthy cats, the clinical dose of beraprost suppresses PAC, which can be reversed by the inhibition of NOS.

## 1. Introduction

Aldosterone and the mineralocorticoid receptor play an essential role in the hemodynamics [1,2,3] and pathomechanisms of feline kidney disease [4]. From substantial and ever-increasing evidence in laboratory animals and humans, chronic kidney disease (CKD) can be considered as a state of relative hyperaldosteronism [2,3]. In cats with hypertensive CKD, increased plasma aldosterone levels have been reported [5,6,7]. Additionally, crosstalk effects between aldosterone and endothelium-derived NO have been experimentally demonstrated in vascular function [8,9].

Beraprost is a chemically and metabolically stable prostacyclin analogue with high and long-lasting oral activity [10]. Beraprost induces endothelial NO synthase (eNOS), increases levels of intracellular NO [11], and also mediates endothelium-independent vasodilation [12,13]. In rats, beraprost has been shown to protect the kidney endothelium and microvasculature, to slow down loss of kidney function [14], and to prolong survival in rodent models of CKD [15]. In a randomized, double-blind, placebo-controlled trial, 74 feline patients with IRIS CKD stage 2 and 3 were recruited and assigned to either beraprost (*n* = 36) or the placebo (*n* = 38) for a period of six months. Compared to the placebo, beraprost slowed kidney function decline, assessed by the change in serum creatinine, without any adverse effects [16]. A retrospective cohort study of 134 cats with IRIS CKD stage 3 in real-world practice also showed that beraprost is associated with prolongation in progression-free survival, defined as time to 25% elevation from baseline serum creatinine levels, and overall survival, defined as time to death from any cause [17]; however, the pharmacological mode of action of beraprost on the feline RA axis and hemodynamics are not known. Various complications and co-morbidities often occur in cats with CKD [18,19,20]. For instance, CKD is reported to be frequently the cause of death in cats with cardiomyopathy [21], hyperthyroidism is common in feline patients with CKD [22,23], and these disorders have been considered to affect the RA axis. Cats with cardiomyopathy reportedly have higher PAC and PRC compared to healthy cats [24]. RA axis activation occurs in hyperthyroid cats, but is not associated with the development of hypertension [25]. In addition, treatment-induced activation of the RA axis occurs with use of the calcium channel blocker amlodipine in hypertensive cats [7,24,26], and loop diuretic furosemide in cardiomyopathy cats [24]. Therefore, the prescription of beraprost has been often limited in feline CKD patients concurrent with the above conditions due to the unknown effect of beraprost on the RA axis.

The purpose of this placebo-controlled sequential design study in the target species was to describe the pharmacological effects of beraprost when orally administered to healthy, young adult, male and female cats at dose levels used in clinical practice for treatment of feline CKD, and to examine whether NOS inhibition with NG-nitro-L-arginine methyl ester (L-NAME) could reverse these effects.

## 2. Materials and Methods

### 2.1. Subjects

Seven domestic Shorthair cats were obtained from a commercial laboratory animal breeder. Their baseline characteristics are shown in Table 1. The cats were acclimated for at least 4 weeks before study initiation and kept under specified pathogen- free conditions and single housed during the conduct of the study. Room temperatures was maintained at 21 ± 2 °C with 50 ± 20% humidity and 12 h light and dark cycle. Cats were allowed to exercise outside the cages for 30 min once daily and fed with a standard pellet diet Prostage Le Chat (Yeaster, Co., Ltd., Hyogo, Japan) twice daily. For the study assessments, the cats were transferred into portable carriers and kept under quiet conditions. If necessary, the carriers were covered with thin blankets to minimize stress and anxiety. No sampling lines and sedative agents were used thus minimizing sampling artifacts.

### 2.2. Study Design

This was a sequential placebo-controlled laboratory study conducted in Tokyo University of Agriculture and Technology, Tokyo, Japan. The use of seven cats for this study was based on availability and practicality, considering the planned study procedures; no attempt was made to estimate an expected effect size, so a priori sample size calculation was not performed. The time schedule in Table 2 describes all study interventions and assessments. All seven cats underwent identical sequential treatments in three study phases, (1) placebo, (2) beraprost, and (3) beraprost + L-NAME. A washout period was not employed between phases but, because each cat received the same treatment sequence in the order described above (placebo, drug, and then drug with inhibitor), carry over effects between phases were not expected to interfere with the study assessments. Furthermore, the chosen design allowed beraprost to reach a steady state in the plasma [27] before the first assessment time point in phase 2, meaning the effects of beraprost evaluated in phase 2 and 3 would be expected to be consistent throughout this period. Study animals were evaluated for body weight, blood pressure, heart rate, and plasma renin and aldosterone levels. An exploratory estimation of renal plasma flow (RPF) and renal vascular resistance (RVR) was made using a simplified para-aminohippuric acid (PAH) clearance method. The same data were collected for each cat twice in each study phase—once in the first and once in the second week of each phase, at approximately the same time each day. Clinical observations were made daily.

### 2.3. Materials

Tablets containing 55 µg beraprost (RAPROS^®^) or placebo were provided in blister packages by Toray Industries, Inc., Tokyo, Japan. Placebo tablets had the same appearance and composition as the beraprost tablets, except that beraprost was replaced by lactose. The tablets were stored according to the manufacturer’s instruction. NG-Nitro-L-arginine methyl ester hydrochloride (L-NAME) was from Fujifilm Wako Pure Chemical Co., Ltd., Tokyo, Japan. It was dissolved in sterile water for injection (10 mg/mL). PAH (10% sodium para-aminohippurate injection) was obtained from Daiichi-Sankyo Co., Ltd., Tokyo, Japan and was diluted in saline for the intravenous injection.

### 2.4. Treatments

Depending on the study phase (Table 2), one tablet per cat of either placebo (Phase 1, Day 1 to 15) or beraprost (Phases 2 and 3, Day 16 to 43) was administered twice daily at feeding. The dosage of beraprost (55 µg per cat, twice daily) corresponds to the dose level which is clinically prescribed for cats with CKD.

In addition, L-NAME was administered once daily on days 36, 37, 42 and 43, starting at the same time that the beraprost tablet was administered in the morning which lasted for 360 min. This administration followed previous the description for NOS inhibition in cats [28]: each cat received L-NAME via intravenous infusion through a peripheral catheter, at a dose rate of 0.1 mg/kg/min (0.01 mL/kg/min).

### 2.5. Methods

Clinical observations for signs of illness, injury, or abnormal behavior were made by a veterinarian according to the Guide for the Care and Use of Laboratory Animals [29]. Body weight was measured using an electronic digital scale accurate to 0.02 kg.

Clinically, beraprost is dosed twice daily in cats. Therefore, the study assessments were targeted at 5 to 6 h (300 to 360 min) after placebo or beraprost tablet administration, to be approximately midway between doses. Mean, systolic and diastolic blood pressures (MBP, SBP, and DBP) and heart rate were measured at 340 min after tablet administration using the oscillometric device petMAP graphic II, Ramsey Medical, Inc., Tampa, FL, USA following the manufacturers’ instruction. All procedures of blood pressure measurements were in accordance with the American College of Veterinary Internal Medicine (ACVIM) consensus statement on management of systemic hypertension in dogs and cats [30].

For determination of PAC and PRC, blood (1.0 mL) was taken from a peripheral vein at 350 min after tablet administration into vials containing anti-coagulant EDTA-2K. Vials were immediately centrifuged at 1510× *g* for 10 min. Plasma samples were kept at −80 °C until determination of PAC and PRC with standard chemiluminescent enzyme immunoassay at Showa Medical Science Co., Ltd., Tokyo, Japan. The limit of detection for PAC and PRC were 25 pg/mL and 0.15 ng/mL, respectively.

**Figure 1:**
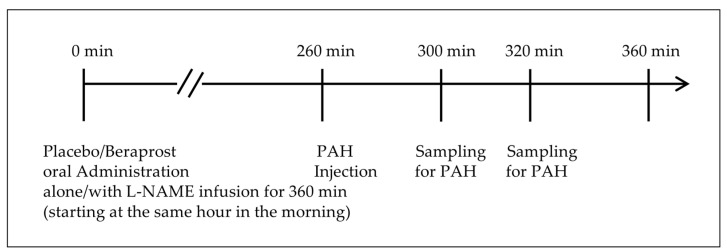
Timeline of treatments and simplified plasma PAH clearance.

A dose of 40 mg/kg (0.4 mL/kg) PAH was administered by bolus intravenous injection at 260 min after placebo or beraprost tablet administration on days 7, 14, 22, 29, 36, and 42. Blood samples (1.2 mL) for determination of PAH clearance were collected via a peripheral vein at 300 and 320 min after tablet administration. These time points were based on the pharmacokinetic profiles in laboratory dogs shown in the product information of PAH provided by Daiichi-Sankyo Co., Ltd. [31]: after a single bolus injection, most of the PAH disappeared from the plasma 30 min to 60 min later. No food was allowed from the time of treatment administration (placebo/beraprost), until completion of plasma PAH clearance testing, to standardize fasting status. The timeline of treatments and plasma PAH clearance on days of assessments is shown in Figure 1. The concentration of PAH was determined by the standard colorimetric method, and the initial concentration (C_0_) and half-value period (T_1/2_) of PAH using the 1-compartment model built from the measured data. This non-corrected 1-compartment model from plasma measurements alone is different from previously reported methods in cats with both plasma and urine [32,33], and can therefore only be considered an exploratory analysis. Using these exploratory PAH clearance values, RPF was estimated by the following formula: RPF = 40 (mg/kg) × Body weight (kg) × loge2 ÷ [C_0_ (mg/mL) × T_1/2_ (min)]. Although renal artery pressure was not assessed, RVR was estimated by the following formula using MBP instead: estimated RVR = MBP/RPF.

### 2.6. Data Management

Data were collected after treatment from a total of seven cats, each providing two sets of data for each study phase as technical replicates with independent experiments to limit the random noise associated with protocols or equipment [34]. 

PAC and PRC [pg/mL and ng/mL], MBP (mmHg), heart rate (bpm), estimated RPF (mL/kg/min), and estimated RVR (mmHg-min/mL) were presented as dot plots of the averaged technical replicates (*n* = 7), and their mean ± standard error (SE). Body weight, SBP, and DBP (mmHg) were described as mean ± SE. PAC and PRC values below the limit of detection were set to half of the limit value.

Data normality was tested using the Shapiro–Wilk test, and appropriate parametric or non-parametric tests were subsequently applied. The differences among three phases: placebo, beraprost, and beraprost combined with L-NAME (NOS inhibitor), were tested by the one-way repeated measures ANOVA followed by the Holm–Bonferroni-corrected *t* test. Effects were calculated using differences between the placebo and beraprost phases for beraprost, and between the beraprost and beraprost + L-NAME for added L-NAME, respectively. Data were presented in mean ± SE. Correlation ratios (η^2^) were obtained and tested by the one-way repeated measures ANOVA. A commercial statistical software program was used for all the analyses (BellCurve for Excel version 3.2; Social Survey Research Information Co., Ltd., Tokyo, Japan). Statistical significance was set at the 2-sided level * *p* < 0.05, ** *p* < 0.01.

## 3. Results

### 3.1. Clinical Observations and Body Weight

All animal procedures were completed according to the study plan, and cats maintained stable clinical condition over the course of the study. Mean body weight [kg] ± SE was 3.22 ± 0.17 in the placebo phase, 3.17 ± 0.17 in the beraprost phase, and 3.21 ± 0.17 in the beraprost + L-NAME phase. Body weight was stable during the study period.

### 3.2. PAC and PRC

Mean aldosterone plasma concentration [pg/mL] ± SE was 79.2 ± 10.9 in the placebo phase, 48.1 ± 8.2 in the beraprost phase and 110.4 ± 9.0 in the beraprost + L-NAME phase. Thus, aldosterone levels were lowered to statistically significant (*p* < 0.05) amounts through treatment with beraprost, which was fully reversible (*p* < 0.01) when adding L-NAME to the beraprost treatment.

**Figure 2:**
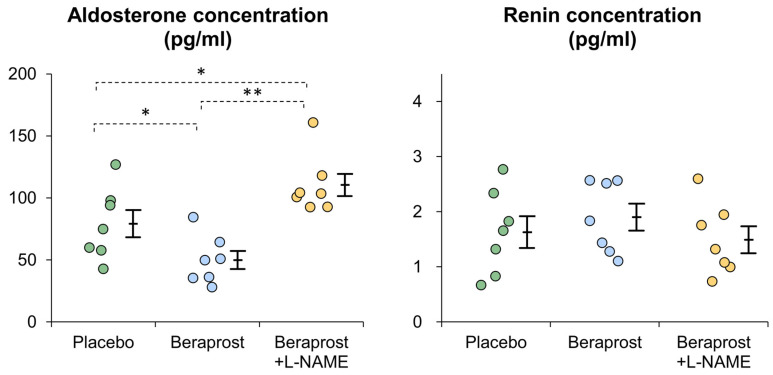
Comparison of plasma aldosterone and renin concentrations (PAC and PRC) in cats administered placebo, beraprost, and beraprost combined with L-NAME (NOS inhibitor). Data represent dot plots for each phase (dot color), mean ± SE of seven cats. * *p* < 0.05, ** *p* < 0.01 by one-way repeated measures ANOVA followed by Holm–Bonferroni-corrected *t* test.

Mean renin plasma concentration [ng/mL] ± SE was 1.63 ± 0.29 in the placebo phase, 1.90 ± 0.24 in the beraprost phase and 1.49 ± 0.25 in the beraprost + L-NAME phase. PRC were similar between all three phases with no significant difference (Figure 2).

### 3.3. Measurements of Hemodynamics

**Figure 3:**
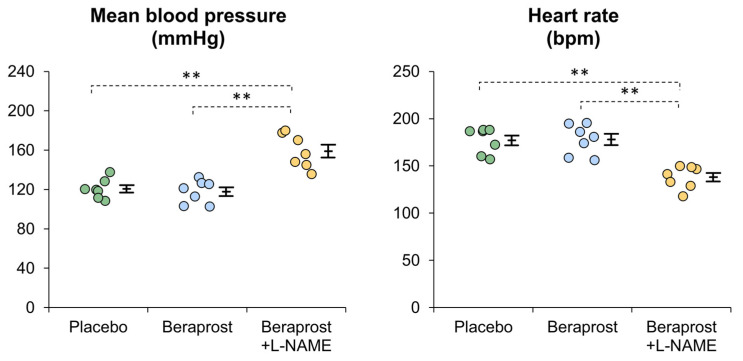
Comparison of mean blood pressure (MBP) and heart rate in cats administered placebo, beraprost, and beraprost combined with L-NAME (NOS inhibitor). Data represent dot plots for each phase (dot color), mean ± SE of seven cats. ** *p* < 0.01 by one-way repeated measures ANOVA followed by Holm–Bonferroni-corrected *t* test.

Mean MBP [mm Hg] ± SE was 120.7 ± 3.7 in the placebo phase, 117.8 ± 4.5 in the beraprost phase and 159.0 ± 6.5 in the beraprost + L-NAME phase. MBP was similar between the placebo and beraprost phases, while it was higher in the beraprost + L-NAME phase than other phases (*p* < 0.01) (Figure 3). In addition, mean SBP [mm Hg] ± SE was 169.2 ± 4.8 in the placebo phase, 170.6 ± 6.3 in the beraprost phase and 217.4 ± 9.3 in the beraprost + L-NAME phase; mean DBP [mm Hg] ± SE was 96.3 ± 4.2 in the placebo phase, 92.4 ± 3.5 in the beraprost phase and 127.6 ± 7.3 in the beraprost + L-NAME phase. These results were similar to those of MBP (dot plots are not shown).

Mean heart rate [bpm] ± SE was 177.1 ± 5.2 in the placebo phase, 178.0 ± 6.0 in the beraprost phase and 138.1 ± 4.5 in the beraprost + L-NAME phase. Heart rate was similar between the placebo and beraprost phases, while it was lower in the beraprost + L-NAME phase (*p* < 0.01) (Figure 3).

In addition, estimated RPF and RVR were exploratorily assessed with simplified methods. Mean estimated RPF [mL/kg/min] ± SE was 11.3 ± 0.8 in the placebo phase, 12.4 ± 0.7 in the beraprost phase and 9.3 ± 0.8 in the beraprost + L-NAME phase. Estimated RPF did not differ significantly between the placebo and beraprost phases, while it was lower in the beraprost + L-NAME phase (*p* < 0.01 vs. beraprost, *p* < 0.05 vs. placebo). Mean estimated RVR [mmHg-min/mL] ± SE was 3.53 ± 0.34 in the placebo phase, 3.09 ± 0.17 in the beraprost phase and 5.94 ± 0.88 in the beraprost + L-NAME phase. Estimated RVR did not differ significantly between the placebo and beraprost phases, while it was higher in the beraprost + L-NAME phase (*p* < 0.01) (Figure S1).

### 3.4. Assessments of the Effects of Beraprost and Added L-NAME (NOS Inhibitor)

In order to compare the effects of beraprost and added L-NAME on PAC and PRC, and hemodynamics, effects were calculated using differences between the placebo and beraprost phases for beraprost, and between the beraprost and beraprost + L-NAME for added L-NAME, respectively. The effects, correlation ratios, and *p*-values are presented in Table 3. The effects of beraprost on PAC and PRC and hemodynamic parameters were counteracted by added L-NAME (*p* < 0.001 for PAC, MBP, heart rate, estimated RPF, *p* < 0.01 for estimated RVR, *p* < 0.05 for PRC).

## 4. Discussion

Results of this study in healthy cats show that oral administration of the clinical dose of beraprost used for feline CKD reduces PAC and this effect is reversed by NOS inhibition with L-NAME. These findings indicate that beraprost may pharmacologically enhance NOS activity in cats. CKD has been associated with impaired bioavailability of NO and endothelial dysfunction. In feline CKD, endogenous eNOS inhibitor asymmetric dimethylarginine (ADMA) is increased in the plasma [35]. Endothelial cell loss detected by endothelial marker CD31 is present in feline kidneys and is positively correlated with kidney dysfunction [36]. Beraprost has a protective effect on injured human umbilical vein endothelial cells (HUVECs) [37], restores impaired endothelial dysfunction in diabetes mellitus rats [12], and induces eNOS and elevated NO production in various animal endothelial cells to improve endothelial function [11]. Our present findings support the assumption that the pharmacological mechanism of beraprost on feline CKD may be endothelial protection through its activation of eNOS and NO production.

Previous studies have described the role of NO in the regulation of aldosterone synthesis. The NO donors including nitroprusside inhibit angiotensin II, potassium, and ACTH-induced aldosterone synthesis in adrenal cells [38,39]. On the contrary, NOS inhibitors cause aldosterone synthesis in adrenal cells [40,41], and increase serum aldosterone in humans [42]. Therefore, attending to the results of the present study in cats with the observed effects of beraprost decreasing plasma aldosterone levels, it can be hypothesized that beraprost modulates aldosterone synthesis in cats via increased NO production. At the same time, clinical dosages of beraprost did not affect plasma renin levels in healthy cats. This was unexpected as beraprost is a synthetic analog of the prostaglandin prostacyclin, and there are previous reports about effects of prostaglandins on renin secretion. In vitro experiments in isolated rat kidneys and glomeruli indicate that prostaglandins stimulate renin secretion [43,44]. 

In human pulmonary hypertension, beraprost above a certain dose exerted a short-term hemodynamic effect [45], and the human maximal tolerated dose of beraprost, four times a day, is reported to improve exercise capacity and symptoms [46]. In feline patients with CKD there were no reports of hemodynamic-related adverse events in two studies using normal clinical dosages [16,17]. With an overdose of ≥30 µg, beraprost/kg bodyweight of healthy cats had increased heart rate within the reference range, without a significant change in blood pressure [47]. In the present study, no biologically or statistically relevant changes in hemodynamic parameters were observed in the beraprost-only phase (slight decrease in MBP (mean ± SE, −2.8 ± 3.5 mmHg) and increase in heart rate (mean ± SE, +0.9 ± 2.1 bpm)). These may be associated with baroreflex control. Together, the present finding that the clinical dose of beraprost for feline CKD did not show a change in hemodynamic parameters may be helpful for clinical practice regarding expected hemodynamic effects.

Loss of peritubular capillaries and tubulointerstitial fibrosis are thought to occur through the tubulointerstitial injury, which increases RVR and further decreases RPF in humans and rodents [48,49]. Similarly in cats with CKD, RPF is reported to be decreased due to kidney injury [50]. Beraprost maintains kidney microvasculature and RPF in rat models of CKD [14]. In a study in human patients with chronic glomerulonephritis, beraprost is shown to prevent a decrease in RPF without increasing the glomerular filtration rate and ratio of afferent to efferent resistance [51]. The present data shows that beraprost has no significant effects on the estimated RPF and RVR in healthy cats (slight increase in estimated RPF [mean ± SE, +1.2 ± 0.4 mL/kg/min] and decrease in estimated RVR [mean ± SE, −0.4 ± 0.2 mmHg-min/mL]). As for the next steps, further evaluation would be beneficial to verify if beraprost prevents decreases in RPF in cats with CKD, and also to examine pharmacokinetics in both healthy and CKD cats.

The effects of L-NAME on hemodynamics were previously examined in two studies with anesthetized cats with inconsistent findings; Marcel van Gelderen et al. (1993) reported that L-NAME did not modify systemic hemodynamic variables [52], while Brown (1993) demonstrated that L-NAME increased systemic arterial pressure and RVR [28]. Brown suspected that the inconsistency might have resulted from the different types of anesthetics used: ketamine vs. pentobarbitone [28]. In this study, L-NAME reversed the effects of beraprost on plasma aldosterone. L-NAME given with beraprost affected hemodynamics, with an increase in blood pressure and estimated RVR, and a reduction of heart rate and estimated RPF; as the pharmacology of L-NAME alone in healthy awake cats remained unexplored, which is a limitation, the significance of this finding is unknown. 

There were several study limitations. A within-subjects repeated measures design, as used in this study, can be at risk for certain biases. Carryover effects cannot be excluded, as no washout periods were conducted between study phases. Whilst this would not be relevant after the first phase (placebo), it cannot be ruled out that beraprost accumulated in the third phase (beraprost + L-NAME), compared to the second (beraprost alone). Practice effects were minimized through long enough acclimation before the study started. In addition, due to the sequential phase design, the study was not randomized, and there was no blinding. It would be ideal to reevaluate the findings in this study with a blinded, randomized design, employing a suitable washout period between phases. Another limitation in this study is that all collected data were from a single time point during an interval of 300 to 360 min after oral administration of beraprost. There are two reasons for this: first, the 300 to 360 min post-dose assessment timing is approximately midway between two doses and, based on in vitro pharmacology and pharmacokinetic data published in the product monograph [27], was thought to be appropriate for measuring the effects of beraprost under its intended repeated dosing schedule (the clinical dosing of beraprost for feline CKD is twice daily, long term); second, this study design involved several measurements in parallel and repetition of measurements that had to be limited for reasons of practicality. 

In addition to the above, PAH clearance was only evaluated on an exploratory basis with a non-corrected, 1-compartment model from plasma measurements alone. This is different from previously reported methods in cats with both plasma and urine [32,33]. The validity of this model is therefore uncertain, so RPF results can also only be considered exploratory. However, the rationale of applying this exploratory approach was based on previous human research on intravenous single bolus methods for plasma PAH clearance [53]. There were also practical reasons: the purpose of the present study was to examine the pharmacological effects of beraprost when orally administered to conscious cats, which could help to better understand the clinical safety of beraprost in feline CKD. The previously described complete methods of PAH clearance are more invasive, requiring anesthesia, and were therefore considered inappropriate for this study due to technical and ethical limitations. However, the present data on estimated RPF in the placebo-phase (mean ± SE, 11.3 ± 0.8 mL/kg/min) and beraprost phase (mean ± SE, 12.4 ± 0.7 mL/kg/min) were similar to previous ones: range from the minimum to maximum, 12.0 to 18.1 mL/kg/min [32], and mean ± standard deviation, 15.1 ± 3.5 mL/kg/min [33]. There also remains room for improvement by possibly referencing the correction such as the Brochner-Mortensen equation for measurement of glomerular filtration rate, as reported in humans [54]. 

Finally, it was previously reported that plasma renin activity in neutered cats is lowered compared to intact cats [55]; therefore, it is not certain if the present findings on PRC from intact cats would fully reflect the effects in neutered cats.

## 5. Conclusions

In healthy cats, the dose of beraprost used clinically for feline CKD resulted in a reduction in PAC, which was reversed by NOS inhibition. Beraprost did not affect PRC, nor hemodynamic parameters. These findings contribute to our understanding of the pharmacological mechanism and clinical safety of beraprost in feline CKD with disorders related to the RA axis and hemodynamics.

## Figures and Tables

**Table 1:** Baseline characteristics of all cats.

No.	Age (years)	Weight (kg)	Sex	Breeds
1	3.4	2.7	Intact Female	Domestic Shorthair
2	3.7	2.6		
3	3.4	3.1		
4	3.4	3.0		
5	2.0	3.8	Intact Male	
6	3.4	2.8		
7	2.0	3.6		

**Table 2:** Time schedule of study procedures.

	Phase 1					Phase 2					Phase 3			
	Placebo					Beraprost					Beraprost + L-NAME			
	Day 1	Day 7	Day 8	Day 14	Day 15	Day 16	Day 22	Day 23	Day 29	Day 30	Day 36	Day 37	Day 42	Day 43
Treatments														
Placebo administration (oral)	Day 1 to 15 inclusive													
Beraprost administration (oral)						Day 16 to 43 inclusive								
L-NAME administration (intravenous)											X	X	X	X
Assessments														
Clinical observations	Day 1 to 43 inclusive													
Body weight		X		X			X		X		X		X	
Plasma PAH clearance		X		X			X		X		X		X	
Blood pressure and heart rate			X		X			X		X		X		X
Plasma aldosterone and renin			X		X			X		X		X		X

X: implementation of treatments or assessments on that study day.

**Table 3:** Effects of beraprost and added L-NAME (NOS inhibitor) and their correlation ratios.

	Aldosterone (pg/mL)	Renin (ng/mL)	Mean Blood Pressure (mmHg)	Heart Rate (bpm)	Estimated Renal Plasma Flow (mL/kg/min)	Estimated Renal Vascular Resistance (mmHg-min/mL)
Beraprost	−31.1 ± 12.0	+0.3 ± 0.2	−2.8 ± 3.5	+0.9 ± 2.1	+1.2 ± 0.4	−0.4 ± 0.2
Added L-NAME	+62.3 ± 10.0	−0.4 ± 0.2	+41.1 ± 6.3	−39.9 ± 2.6	−3.2 ± 0.6	+2.9 ± 0.8
Correlation ratio (η^2^)	0.749	0.357	0.756	0.926	0.758	0.593
*p*-value	** p < 0.001	0.0241 *	** *p* < 0.001	** *p* < 0.001	** *p* < 0.001	0.0013 **

Data represent mean difference ± SE from placebo of seven cats. * *p* < 0.05, ** *p* < 0.01 by one-way repeated measures ANOVA.

## Data Availability

The raw data supporting the conclusions of this article will be made available by the authors, without undue reservation.

## Supplementary Materials

The following supporting information can be downloaded at: https://www.mdpi.com/article/10.3390/vetsci11040155/s1, Figure S1: Comparison of estimated renal plasma flow and renal vascular resistance in cats administered placebo, beraprost, and beraprost combined with L NAME (NOS inhibitor).
