# Supplementary material for: Effects of Beraprost with or without NOS Inhibition on Plasma Aldosterone and Hemodynamics in Healthy Cats
Source: Vet Sci. 2024 Mar 30;11(4):155. doi: 10.3390/vetsci11040155 (PMC11054574; doi:10.3390/vetsci11040155)
Supplement: Supplementary file 1 [file vetsci-11-00155-s001.zip › vetsci-2879672-supplementary.pdf]

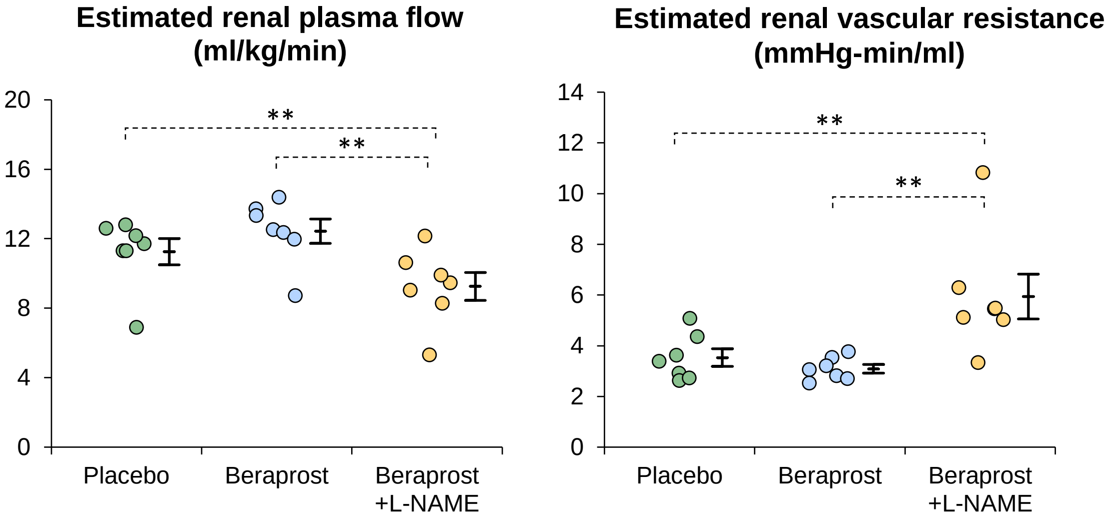


**Figure S1.** Comparison of estimated renal plasma flow and renal vascular resistance in cats administered placebo, beraprost, and beraprost combined with L-NAME (NOS inhibitor). Data represent dot plots for each phase (dot color), mean ± SE of seven cats * $p < 0.05$, ** $p < 0.01$ by one-way repeated measures ANOVA followed by Holm–Bonferroni-corrected t test.
